# Antioxidant activity and components of a traditional chinese medicine formula consisting of *Crataegus pinnatifida* and *Salvia miltiorrhiza*

**DOI:** 10.1186/1472-6882-13-99

**Published:** 2013-05-10

**Authors:** Cheng-Yu Chen, Hua Li, Ya-Nan Yuan, Hui-Qing Dai, Bin Yang

**Affiliations:** 1 Institute of Chinese Materia Medica, China Academy of Chinese Medical Sciences, Beijing, China

**Keywords:** Traditional Chinese medicine, *Crataegus pinnatifida*, *Salvia miltiorrhiza*, Antioxidant activity, Antioxidant compounds, HPLC, Oxygen radical absorbance capacity, DPPH assay

## Abstract

**Background:**

The aims of this study were to evaluate the antioxidant activity and to identify the antioxidant components of a traditional Chinese medicine formula consisting of a combination of Shanzha (the fruit of *Crataegus pinnatifida* Bge. var. *major* N.E.Br., SZ) and Danshen (the root of *Salvia miltiorrhiza* Bge., DS). This medicine is extensively used to treat cardiovascular disease.

**Methods:**

Twelve samples extracted and fractionated from SZ, DS and the formula (SZ+DS) were analyzed. The concentrations of eight phenolic compounds were determined by high performance liquid chromatography. Oxygen radical absorbance capacity (ORAC) assay and 1,1-diphenyl-2-picrylhydrazyl assay were conducted to explore the antioxidant activities of the samples and of the 15 phenolic compounds detected. Correlation analysis of the antioxidant activity of herb samples and their phenolic components was performed.

**Results:**

The main phenolic component in all SZ+DS samples was salvianolic acid B, which exhibited strong antioxidant activity (ORAC value: 16.73 ± 2.53, IC50 value: 8.80 ± 0.06 μM) compared with the other phenolic compounds. For all samples, there was a positive relationship between their total phenolic components and their antioxidant activities.

**Conclusions:**

Phenolic compounds were the bioactive components of the herb samples, and salvianolic acid B was identified as the main bioactive compound in the SZ+DS formula.

## Background

Shanzha (the fruit of *Crataegus pinnatifida* Bge. var. *major* N.E.Br., SZ) and Danshen (the root of *Salvia miltiorrhiza* Bge., DS) are medicinal plants used extensively in traditional Chinese medicine (TCM). These two herbs (SZ+DS) are used together to treat cardiovascular diseases such as coronary disease and hyperlipemia. DS promotes blood circulation and tissue regeneration, while SZ prevents thrombosis and promotes digestion. In TCM, the combination of these two herbs is said to promote the qi circulation, nourish blood, remove blood stasis, and relieve pain [1]. Numerous studies have shown that cardiovascular disease is the result of a metabolic imbalance of endogenous radicals [2], so it is possible to treat it by administering antioxidants.

Both SZ and DS exhibit high antioxidant activities [3,4]. The active components of SZ are mainly phenolic compounds such as chlorogenic acid, rutin, and (−)-epicatechin [5], while the active components of DS have been identified as salvianolic acid A and salvianolic acid B [6]. However, knowledge about the antioxidant capacity and the bioactive components in the SZ+DS combination is scarce. In most cases, the chemical ingredients in a herbal formula differ from those found in the individual herbs. Therefore, it is necessary to investigate the antioxidant activities and active components of the formula.

Various *in vitro* methods have been used to measure the antioxidant capacity of samples. According to the mechanism by which antioxidants quench radicals, these methods are divided into two clusters. One is based on the ability of an antioxidant to deactivate radicals by hydrogen donation, and includes oxygen radical absorbance capacity (ORAC) assay, total oxidant scavenging capacity assay, and low-density lipoprotein assay. The other refers to the capacity of an antioxidant to terminate radicals through electron transferring, and includes 1,1-diphenyl-2-picrylhydrazyl (DPPH) assay and Trolox equivalent antioxidant capacity assay [7].

In this paper, we investigated the antioxidant components in SZ+DS by using an High-performance liquid chromatography (HPLC)-based analytical method together with assays to measure antioxidant capacities. The HPLC method was used to characterize and quantify the phenolic components contained in the SZ, DS, and SZ+DS extracts, while the antioxidant assays were conducted to provide a comprehensive analysis of the antioxidant activities of the extracts and their phenolic components. Furthermore, we performed a correlation analysis of the antioxidant activities of the extracts and their corresponding phenolic components.

## Methods

### Chemicals and plants

Salvianolic acid A and rosmarinic acid were purchased from the National Pharmaceutical Engineering Center for Solid Preparation in Chinese Herbal Medicine (Nanchang, China). Lithospermic acid was obtained from the Shanghai Ronghe Pharmaceutical Company (Shanghai, China). Procyanidin B_2_ was obtained from Chromadex. Salvianolic acid B, sodium danshensu, protocatechuic aldehyde, hyperoside, rutin, and (−)-epicatechin were purchased from the National Institute for Food and Drug Control (Beijing, China). Caffeic acid, 2,2'-azobis (2-methyl- propionamidine) dihydrochloride (AAPH), and DPPH were purchased from Sigma Chemical Co. (St. Louis, MO, USA). Quercetin, isoquercitrin, and fluorescein sodium were purchased from Fluka Chemical Co. Protocatechuic acid was obtained from Wako Pure Chemical Industries, Ltd. (Osaka, Japan). Trolox was purchased from Aldrich. Deionized water was prepared using a Milli-Q water purification system (Millipore, France). HPLC grade acetonitrile was purchased from Burdick & Jackson (USA). HPLC grade tetrahydrofuran was obtained from Fisher (USA). Other reagents were of analytical grade. DS and SZ were collected from Shandong Province (China) and identified by the authors as the fruit of *C. pinnatifida* var. *major* and the root of *S. miltiorrhiza*, respectively. Voucher specimens were deposited in the herbarium of the Institute of Chinese Materia Medica, China Academy of Chinese Medical Sciences, Beijing, China. HPLC analysis was performed on a Waters Alliance 2695 System equipped with a Waters 2996 DAD. ORAC and DPPH assays were performed on a Varioskan Flash Multimode Reader (Thermo Scientific, Finland).

### Sample preparation

DS was reflux extracted for 1 h with a 10-fold water volume. The extract was filtered and centrifuged at 4000 rpm for 10 min, and the supernatant (DS water extract, DSW) was concentrated and dried up to produce the concentrated DS water extract 1 (DSW 1). Ethyl acetate was used to separate the lipophilic ingredients from the aqueous solution of DSW 1; both phases were concentrated to dryness and named as DS ethyl acetate extract (DSE) and concentrated DS water extract 2 (DSW 2), respectively. The SZ and SZ+DS samples were prepared in the same way, consequently obtaining eight extracts: SZ water extract (SZW), concentrated SZ water extract 1 (SZW 1), SZ ethyl acetate extract (SZE), concentrated SZ water extract 2 (SZW 2), SZ+DS water extract (SDW), concentrated SZ+DS water extract 1 (SDW 1), SZ+DS ethyl acetate extract (SDE), and concentrated SZ+DS water extract 2 (SDW 2).

### HPLC analysis

HPLC analysis was carried out on an Agilent Extend C18 (4.6 × 250 mm, 5 μm) column. The mobile phase consisted of 50 mM phosphate buffer (pH 2.5) (solvent A), and methanol-acetonitrile-tetrahydrofuran (10:90:0.05, V/V/V) (solvent B). A gradient program was adopted for the analysis. The flow rate was 1.0 ml/min. The column temperature was set at 30°C. All concentrated water extract samples were extracted with 20 mL of 70% methanol in a reflux bath for 30 min. After cooling, the obtained solution was adjusted to the original weight, and filtered through a membrane filter (0.22 μM) before injection. All ethyl acetate extract samples were dissolved in 10 ml of 70% methanol and filtered through a membrane filter (0.22 μM) before injection. All water extract samples were filtered through a membrane filter (0.22 μM) directly before injection. The injection volume was 10 μl.

### ORAC assay

ORAC assay was carried out using our previously reported method [8]. Briefly, the reaction mixture was prepared with 100 μl of 0.08 nM fluorescein sodium in phosphate buffer solution (PBS) (75 mM, pH 7.4) and 50 μl of sample in 96-well plates. After incubation at 37°C for 30 min, 50 μl of 153 mM AAPH solution in PBS was added. The fluorescence (λ_excitation_ = 490 nm; λ_emission_ = 514 nm) of each well was measured at 1 min intervals. A standard curve of Trolox was plotted (10–100 μM) with each measurement. The antioxidant capacity was calculated based on the area under the fluorescence curve. The capacity was expressed as Trolox equivalents (μM trolox/μM compound, μM trolox/g extract).

### DPPH assay

Scavenging of DPPH radical was determined according to our previously reported method with few modifications [9]. Briefly, 100 μl of each sample, dissolved in methanol, was poured into 96-well plates and 150 μl of 130 μM DPPH was added. The absorbance of the reaction mixture at 516 nm was continuously measured at 1 min intervals until the differences between data were within 0.003.

## Results and discussion

### Method validation

Method validation was performed on parameters such as linearity, precision and recovery. Calibration curves were constructed from peak areas versus compound amounts. The limit of detection (LOD) and limit of quantification (LOQ) for each marker compound were determined at signal-to-noise ratios of 3 and 10, respectively. The calibration data are shown in Table 1. Relative standard deviation (RSD) values were calculated and considered as the measure of precision. To assess the intra-day precision, the standard solution was injected eight times within a day. The inter-day precision test was assessed by testing over three consecutive days (Table 2). The accuracy of quantitation in terms of recovery was assessed. Standard solutions were spiked into the sample solution containing half the mass of the plant material with known amounts of the tested analytes, and the sample was extracted according to the procedure described in Sample preparation. The amount of each analyte in the standard solution was almost equal to that in the sample solution. The average recovery rate of the eight analytes ranged from 97.74% to 103.38%, with RSD values varying between 1.32% and 5.91% (Table 2).

**Table 1:** Calibration curves, LOD and LOQ of the investigated compounds

**Analyte**	**Calibration curve**	**Linear range/μg**	***r***	**LOD/ng**	**LOQ/ng**
Danshensu sodium	*Y*=547569*X-*21458	0.09-6.80	0.9993	39.0	97.6
Protocatechuic aldehyde	*Y*=4413435*X*-17209	0.02-2.31	0.9999	2.0	20.0
Rosmarinic acid	*Y*=2057826*X*+15905	0.03-3.60	0.9995	8.6	34.6
Lithospermic acid	*Y*=1114788*X*-45648	0.08-8.00	0.9999	33.9	84.8
Salvianolic B	*Y*=1113657*X-*91003	0.04-10.80	0.9998	10.7	42.8
Salvianolic A	*Y*=2744138*X*-38060	0.03-4.50	0.9997	6.0	30.0
Procyanidin B_2_	*Y*=630617*X*+1435	0.07-1.60	0.9996	25.0	75.0
(−)-Epicatechin	*Y*=682521*X*-3363	0.05-1.80	0.9997	14.0	56.0

**Table 2:** Precision and recovery for the developed HPLC method

**Analyte**	**Precision**		**Recovery**	
	**Intra-day variability RSD (%)**	**Inter-day variability RSD (%)**	**Average (%)**	**RSD (%)**
Danshensu sodium	3.33	3.77	98.77	1.64
Protocatechuic aldehyde	3.37	3.82	97.74	1.99
Rosmarinic acid	3.29	3.23	100.22	2.32
Lithospermic acid	2.57	2.61	101.21	1.32
Salvianolic B	3.48	0.73	103.38	3.34
Salvianolic A	3.27	3.45	99.19	2.56
Procyanidin B_2_	2.27	2.82	100.00	5.40
(−)-Epicatechin	2.57	2.54	98.85	5.91

### Sample analysis

**Figure 1:**
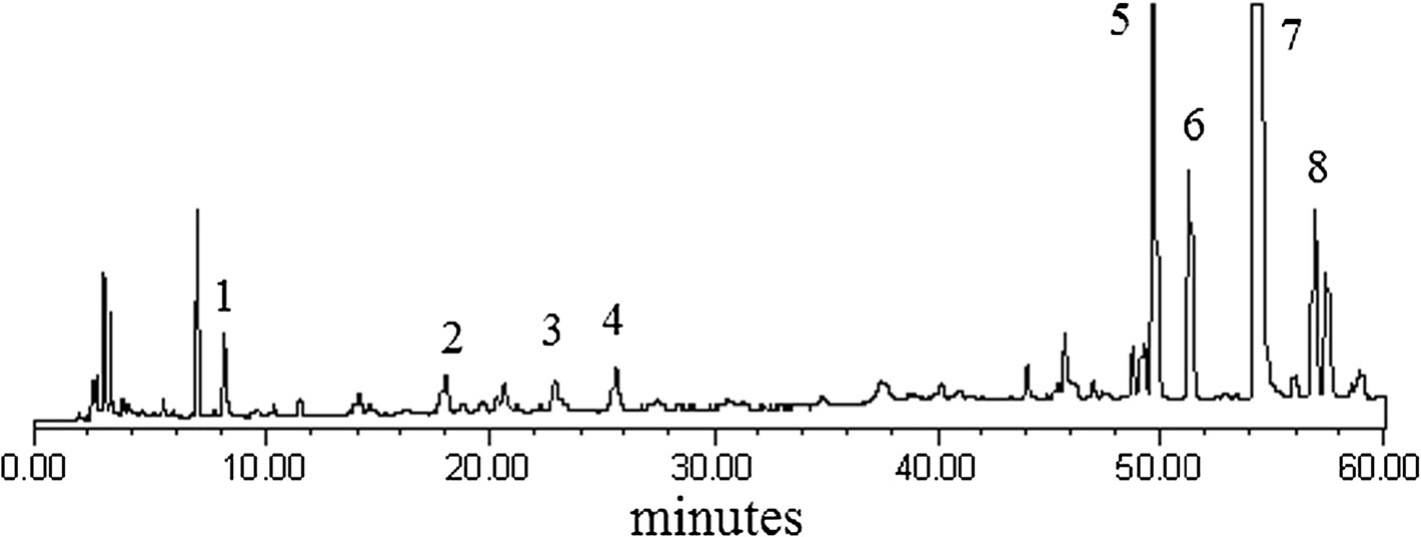
**HPLC chromatogram of SDW at 288nm.** 1, danshensu; 2, chlorogenic acid; 3, procyanidin B2; 4, (−)-epicatechin; 5, rosmarinic acid; 6, lithospermic acid; 7, salvianolic B; 8, salvianolic A.

Eight phenolic compounds were identified from all samples of SZ, DS, and SZ+DS by comparing their retention times and UV spectra with those of standard compounds (Figure 1).

**Figure 2:**
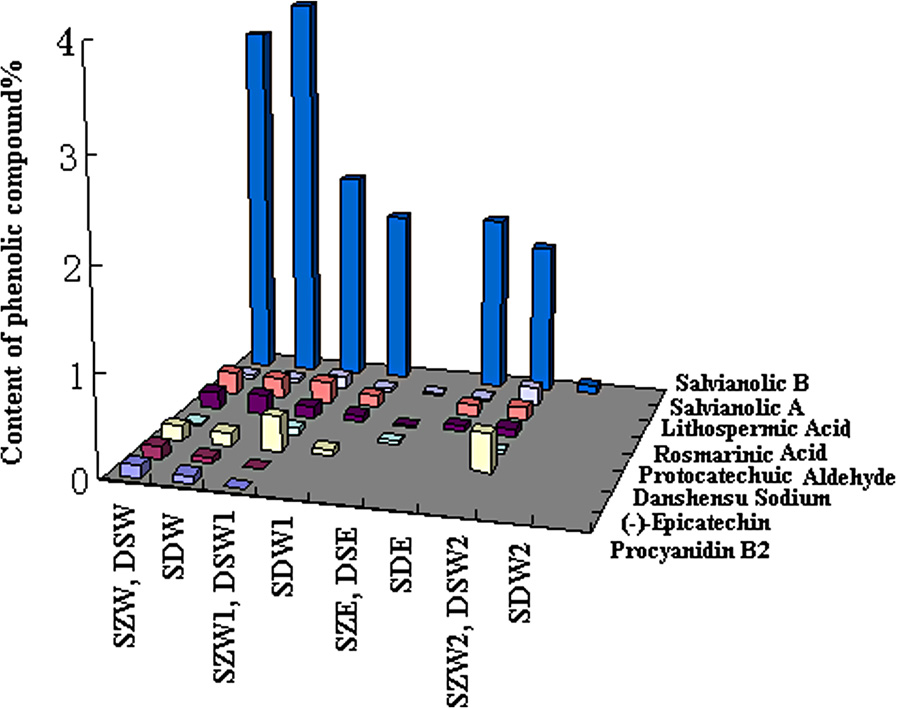
Phenolic components in all herb samples.

Most phenolic components found in SZ and DS were also detected in the SZ+DS formula, except for protocatechuic aldehyde. SDW had a lower content of procyanidin B_2_ and (−)-epicatechin compared with SZW, and a slightly higher content of salvianolic acid B compared with DSW. The amounts of danshensu, rosmarinic acid, lithospermic acid, salvianolic acid B and salvianolic acid A identified in DSW 1 were higher than those in SDW 1. Compared with DSE, SDE had higher contents of rosmarinic acid, lithospermic acid, and salvianolic acid B. The amount of salvianolic acid B in SDW 2 was lower than that in DSW 2. Regarding total phenolic compounds, SDW had the highest content, followed by DSW, while SZ samples had the lowest phenolic content (Table 3 and Figure 2).

**Table 3:** Content of phenolic compounds in herb samples/% (n=2)

	**Danshensu**	**Protocatechuic aldehyde**	**Rosmarinic acid**	**Lithospermic acid**	**Salvianolic B**	**Salvianolic A**	**Procyanidin B**_**2**_	**(−)-Epicatechin**
DSW 1	0.34	0.06	0.12	0.21	2.07	0.11	—^*^	—^*^
DSE	—^*^	0.03	0.01	—^*^	—^*^	0.02	—^*^	—^*^
DSW 2	0.37	0.03	0.08	0.13	1.48	0.18	—^*^	—^*^
DSW	0.15	0.02	0.17	0.22	3.5	0.05	—^*^	—^*^
SZW 1	—^*^	—^*^	—^*^	—^*^	—^*^	—^*^	0.01	0.01
SZE	—^*^	—^*^	—^*^	—^*^	—^*^	—^*^	—^*^	—^*^
SZW 2	—^*^	—^*^	—^*^	—^*^	—^*^	—^*^	—^*^	—^*^
SZW	—^*^	—^*^	—^*^	—^*^	—^*^	—^*^	0.12	0.12
SDW 1	0.07	—^*^	0.07	0.11	1.70	0.03	—^*^	—^*^
SDE	—^*^	—^*^	0.06	0.11	1.73	0.02	—^*^	—^*^
SDW 2	—^*^	—^*^	—^*^	—^*^	0.16	—^*^	—^*^	—^*^
SDW	0.14	—^*^	0.18	0.2	3.82	0.05	0.07	0.06

*No detected.

Rutin, hyperoside, and isoquercitrin were detected in SZW, but were not found in its concentrated water extract. Quercetin and tanshinone, which are hydrophobic and reported to be present in SZ and DS, respectively, were not detected in our experiment because of the solvent extraction treatment.

No novel chromatographic peak was found in SZ+DS samples. Interestingly, lithospermic acid and salvianolic acid B were detected in SDE, but neither was found in DSE. This might be explained by the high content of organic acid in SZ, which may have lowered the pH value of SDW, contributing to the extraction of both phenolic compounds by ethyl acetate.

### Antioxidant activity of herb samples and phenolic compounds

SDW exhibited the highest DPPH and AAPH free radical scavenging capacity, followed by DSW, SDW 1 and SDE, while SZE had the lowest values. The ORAC value of DS was similar to those reported in the literature [10,11], whereas the ORAC value of SZ differed greatly (190 μmolg^-1^ vs. 1240 μmolg^-1^) [11], which may be explained by the temperature of extraction (Table 4).

**Table 4:** **ORAC value and IC**_**50 **_**value of extract**

	**ORAC value/μmol·g**^**-1**^	**IC**_**50 **_**value/μg·ml**^**-1**^
SZE	28±3	10536.67±514.10
DSE	39±3	2285.71±56.13
SZW 2	83±5	2362.45±90.24
SZW 1	190±31	786.88±17.60
SDW 2	209±19	746.35±16.60
SZW	375±41	412.67±12.77
DSW 2	603±27	198.74±7.36
SDW 1	768±94	178.20±3.52
DSW	757±26	131.15±10.29
DSW 1	788±47	96.39±1.35
SDE	916±48	219.95±24.90
SDW	1130±128	63.43±0.84

The 15 phenolic compounds found in the samples appeared to have strong free radical-scavenging activity, with procyanidin B_2_ and salvianolic acid B showing the highest values and chlorogenic acid the lowest (Table 5). In terms of structure-activity relationship, flavonoids such as rutin, hyperoside, and isoquercitrin, whose C-3 position is glycosylated, exhibited less free radical-scavenging capacity than their corresponding aglycon, quercetin. Flavonol showed higher radical-scavenging capacity than flavan (e.g. quercetin > (−)-epicatechin), while procyanidin B_2_, which consists of two (−)-epicatechin units, had higher antioxidant activity than its monomer. Phenolic acid compounds such as salvianolic acid B, salvianolic acid A, rosmarinic acid, and lithospermic acid, which are composed of danshensu and caffeic acid units, exhibited higher radical-scavenging capacity than their monomer counterparts. Among phenolic compounds, salvianolic acid B was the most powerful radical scavenger, which is consistent with Zhao’s result [12]. In summary, the conjugation system formed through the unsaturated double bond located at C_2_-C_3_ of flavonol compounds and their C-_3_ hydroxyl might contribute to the high radical-scavenging capacity of these compounds. Furthermore, such capacity can also be enhanced through polymerization of flavone or phenolic compounds, owing to the increase of hydroxyl groups.

**Table 5:** **ORAC value and IC**_**50 **_**value of compounds**

	**ORAC value**	**IC**_**50 **_**value/μM**
Chlorogenic acid	3.50±0.36	27.50±0.77
Danshensu sodium	5.30±0.31	23.00±0.26
Caffeic acid	5.31±0.18	23.60±0.47
Protocatechuic aldehyde	6.03±0.90	13.30±0.26
Hyperoside	6.20±1.08	22.00±0.19
Rosmarinic acid	7.02±1.03	13.50±0.10
Protocatechuic acid	7.84±1.26	17.50±0.25
Isoquercitrin	7.86±0.67	18.83±0.05
Rutin	9.62±1.40	18.30±0.49
Salvianolic A	10.26±0.82	12.10±0.15
(−)-Epicatechin	11.10±0.41	20.40±0.15
Lithospermic acid	11.40±0.71	15.61±1.07
Quercetin	12.90±0.35	13.10±0.26
Salvianolic B	16.73±2.53	8.80±0.06
Procyanidin B_2_	20.29±1.80	11.20±0.30

### Correlation between the antioxidant activity of the samples and their phenolic compounds

A positive relationship was established between the ORAC value of the herb samples and their total phenolic components, being represented with the linear equation *Y* = 530.92 *X* - 495.05, *r* = 0.9845, indicating that the higher the concentration, the higher the ORAC value. Similarly, a negative relationship was found between the IC_50_ value of the herb samples and their total phenolic components, being represented with the linear equation *Y* = −63.207 Ln (*X*) + 211.12, *r* = 0.9424, indicating that the higher the concentration, the lower the IC_50_ value. The content of salvianolic acid B correlated well with the radical-scavenging capacity of the DS and SZ+DS samples, suggesting that it was the main antioxidant component in these samples.

## Conclusions

Our study demonstrated that phenolic compounds exhibited potential antioxidant activities and can be considered as bioactive components of DS and SZ. Among these compounds, salvianolic acid B from DS showed the highest activity and was identified as the main bioactive compound in the SZ+DS formula.

## Competing interests

The authors declare that they have no competing interest.

## Authors' contributions

C-yC was responsible for acquisition of data, analysis, drafting of the manuscript. HL, Y-nY and H-qD made contribution to collection and preparation of herbal samples. BY was coordinator and designed all the assays. All authors read and approved the final manuscript.

## Pre-publication history

The pre-publication history for this paper can be accessed here:

http://www.biomedcentral.com/1472-6882/13/99/prepub
